# Genetically‐Programmed Hypervesiculation of *Lactiplantibacillus Plantarum* Increases Production of Bacterial Extracellular Vesicles with Therapeutic Efficacy in a Preclinical Inflammatory Bowel Disease Model

**DOI:** 10.1002/advs.202512679

**Published:** 2025-11-26

**Authors:** Nicholas H Pirolli, Daniel Levy, Alyssa Schledwitz, Natalia Sampaio Moura, Mitali Sarkar, Talia J. Solomon, Emily H. Powsner, Raith Nowak, Zuzanna Mamczarz, Christopher J. Bridgeman, Sulayman Khan, Andrew Hui, Nidhi Anne, Laura Reus, William E. Bentley, Jean‐Pierre Raufman, Steven M. Jay

**Affiliations:** ^1^ Fischell Department of Bioengineering University of Maryland College Park MD 20742 USA; ^2^ Robert E. Fischell Institute for Biomedical Devices University of Maryland College Park MD USA; ^3^ Department of Medicine Division of Gastroenterology and Hepatology University of Maryland School of Medicine Baltimore MD USA; ^4^ Department of Biochemistry and Molecular Biology University of Maryland School of Medicine Baltimore MD USA; ^5^ Biomedical Laboratory Research and Development Service Veterans Affairs Maryland Healthcare System Baltimore MD USA; ^6^ Marlene and Stewart Greenebaum Comprehensive Cancer Center University of Maryland Medical Center Baltimore MD USA; ^7^ Program in Molecular and Cell Biology University of Maryland College Park MD 20742 USA

**Keywords:** bacterial membrane vesicles, colitis, extracellular vesicles, IBD, lactic acid bacteria

## Abstract

Inflammatory bowel diseases (IBD) affect over 6 million people globally and current treatments achieve only 10‐20% rates of durable disease remission. Bacterial extracellular vesicles (BEVs) from probiotic lactic acid bacteria (LAB) are a promising novel therapeutic with mechanisms holding potential to drive increased rates of durable disease remission, including immunomodulation and intestinal epithelial tissue repair. However, translation of these cell‐secreted nanovesicles is limited by long standing biomanufacturing hurdles, especially low production yields due to low biogenesis rates from cells. Here, Lactiplantibacillus plantarum is identified as a candidate LAB producing BEVs effective in treating acute dextran sulfate sodium (DSS)‐induced murine colitis with greater efficacy than BEVs from probiotic *Escherichia coli* Nissle 1917. Genetic engineering of *L. plantarum* to create a hypervesiculating strain via inducible expression of a peptidoglycan‐modifying enzyme is shown to enable a 66‐fold increase in BEV productivity. Finally, hypervesiculating *L. plantarum* BEVs are confirmed to be therapeutically effective in the acute DSS mouse model of colitis, with superior reduction of mucosal tissue damage compared to live *L. plantarum* cells. These findings demonstrate that BEVs from genetically engineered hypervesiculating strain of *L. plantarum* are a promising preclinical therapeutic candidate for IBD that overcomes historical biomanufacturing limitations of BEV therapeutics.

## Graphical abstract

The development of a novel engineered strain of *Lactiplantibacillus plantarum* (Lp) is reported that can produce bacterial extracellular vesicles (BEVs) at >60‐fold higher yields than the unmodified parental strain. These Lp BEVs retain therapeutic bioactivity as validated in a mouse model of dextran sodium sulfate‐induced colitis. These results represent a crucial biomanufacturing advance that can set the stage for future translation.

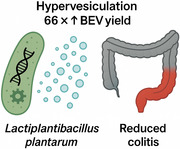

## 1 Introduction

Inflammatory bowel disease (IBD) refers to a group of diseases characterized by multifactorial pathology involving i) profound intestinal inflammation, ii) shifts in microbiome composition, and iii) intestinal tissue damage resulting in loss of gastrointestinal (GI) barrier integrity. The major subtypes of IBD are ulcerative colitis (UC) and Crohn's disease (CD), with over 6 million people afflicted worldwide^[^ ^1^, ^2^ ^]^ resulting in impaired quality of life^[^ ^3^, ^4^, ^5^, ^6^ ^]^ and increased risk of cancer^[^ ^7^ ^]^ and need for surgery.^[^ ^8^ ^]^ All current IBD therapeutics target inflammation; most are not specific to GI inflammation (e.g., anti‐TNFα antibodies or JAK inhibitors), others are specific to GI inflammation (anti‐a4b7 antibodies).^[^ ^9^ ^]^ Regardless of their specificity, these treatments are all characterized by a so‐called “therapeutic ceiling” in which only 10–20% of patients treated with current IBD treatments will achieve and maintain disease remission 1 year after treatment.^[^ ^10^ ^]^ Furthermore, specific safety concerns (infection risk), high costs ($40000 – $150000 per year), and inconvenience (injection or infusion) limit treatment adherence and access to care, further degrading patients’ quality of life. Thus, a paradigm shift in IBD treatment is needed, with increased attention to the multifactorial causes of IBD, particularly environmental (e.g., gut microbiome), genetic (and epigenetic), and immune factors,^[^ ^11^, ^12^, ^13^ ^]^ toward delivery of more effective, safe, affordable, and convenient treatment for patients with IBD.^[^ ^10^ ^]^

Among other biotechnological applications, bacterial extracellular vesicles (BEVs) are cell‐secreted nanovesicles that have emerged as a promising therapeutic modality for IBD.^[^ ^14^ ^]^ BEVs from Gram‐negative bacterial species have been developed as vaccines (Bexsero) but are not suitable for IBD applications due to high pro‐inflammatory lipopolysaccharide (LPS) content.^[^ ^15^ ^]^ On the other hand, BEVs from Gram‐positive species of probiotic/commensal strains found in the human microbiome lack LPS and are effective in preclinical models of IBD,^[^ ^16^ ^]^ as well as other inflammatory diseases. Often, these BEVs are derived from species of Gram‐positive lactic acid bacteria (LAB), such as *Lactobacillus* spp. and *Bifidobacterium* spp. Unlike current IBD therapies, these Gram‐positive LAB BEVs can target novel therapeutic mechanisms to promote intestinal barrier integrity and immunomodulation, while retaining normal immune function, potentially owing to their evolved role as mediators of normal gut microbiome‐host signaling. Thus, via these mechanisms, Gram‐positive LAB BEVs have the potential to overcome therapeutic ceilings and drive more complete mucosal healing, which can promote durable remission of disease and mitigate long‐term risks of cancer and need for surgery.

Despite the promise of Gram‐positive LAB BEVs to improve IBD treatment, to date, their development has been limited due to i) low potency and ii) prohibitively low production yields caused by low biogenesis rates.^[^ ^17^ ^]^ These limitations are related‐low potency necessitates high doses to achieve therapeutic effects, and high dose requirements exacerbate low production yields. Rigorous downstream purification processes can increase potency^[^ ^18^ ^]^ but also result in product loss, whereas more crude methods contribute to batch variability during production, creating a major potential regulatory hurdle. Thus, engineered solutions to improve low yields and increase potency would greatly enable further development and translation of LAB BEV therapeutics for IBD. Toward this end, several approaches to increase BEV yields have been investigated. Environmental modifications or drug treatments have produced only modest two‐ to five‐fold increases in BEV yields and could introduce regulatory concerns depending on the drug treatment used.^[^ ^19^, ^20^, ^21^ ^]^ Genetic deletion of membrane integrity proteins in Gram‐negative bacteria such as *E. coli* can generate orders of magnitude increases in BEV yields, but at the expense of cell viability and BEV bioactivity,^[^ ^22^, ^23^ ^]^ and homologous membrane integrity proteins do not exist in Gram‐positive species of probiotics. Finally, extraction of BEVs from cell pellets using detergents or sonication is effective in Gram‐negative bacteria,^[^ ^24^ ^]^ but Gram‐positive bacteria are highly resistant to such approaches due to their distinct membrane architecture.^[^ ^25^ ^]^ Thus, while large BEV yield increases are possible in general, there is currently no robust system or method for generating meaningful increases in yields of LAB BEVs that might be most effective for IBD treatment.

In this study, we sought to develop such a system by focusing on four LAB species that have shown promising clinical efficacy as live cell formulations. In particular, an 8‐strain combination of LAB (VSL#3) has established clinical efficacy in certain subtypes of IBD, i) mild‐moderate ulcerative colitis, and ii) UC patients after ileal pouch‐anal anastomosis surgery.^[^ ^26^, ^27^, ^28^, ^29^ ^]^ We selected two strains from VSL#3 with clinical efficacy as single‐strain formulations in various GI diseases and symptoms, *Lactiplantibacillus plantarum* ^[^ ^30^, ^31^, ^32^, ^33^ ^]^ and *Lacticaseibacillus paracasei*,^[^ ^34^ ^]^ and two other LAB strains also clinically effective, *Limosilactobacillus reuteri* ^[^ ^35^ ^]^ and *Lacticaseibacillus rhamnosus*.^[^ ^36^ ^]^ We compared BEV characteristics, in vitro anti‐inflammatory activity, and efficacy in an acute DSS mouse model of IBD to identify a suitable cell source for genetic engineering. We then established proof‐of‐concept that LAB species, specifically *L. plantarum*, can be engineered to generate high amounts of BEVs via a genetically programmed hypervesiculation mechanism. We further showed that these high‐yield BEVs retain their effectiveness in the IBD mouse model. Accordingly, the data supports continued preclinical development of hypervesiculating *L. plantarum* BEVs toward the translation of an effective, non‐immunosuppressant, cost‐effective, and convenient treatment for IBD that overcomes the therapeutic ceiling of conventional systemic immunotherapies.

## 2 Experimental Section

### 2.1 Cell Culture

*E. coli* Nissle 1917 (EcN was obtained from Mutaflor (Canada) and DH5a was obtained from New England Biolabs (Boston, MA). All lactic acid bacteria (LAB) strains were obtained through ATCC (*Lacticaseibacillus rhamnosus* GG (ATCC 53 103), *Lacticaseibacillus paracasei* (ATCC 334), *Limosilactobacillus reuteri* F 275 (ATCC 23 272), *L. plantarum* WCFS1 (ATCC BAA‐793).

EcN and DH5a bacterial strains were cultured in LB media at 37 °C at 250 rpm shaking for all experiments. All LAB strains were cultured at 37 °C without shaking (static); for in vitro assays, LAB were cultured in De Man–Rogosa–Sharpe (MRS) media, and for bioactivity experiments, LAB were cultured in complete defined *Lactobacillus* media (LDMIIG) as in prior studies^[^ ^37^ ^]^ except with two modifications: 1 g L^−1^ of Tween 80 was included and tryptophan increased to 0.2 g L^−1^.

### 2.2 BEV Production

An overnight culture was started from a single colony. The overnight culture was then used to inoculate fresh media to commence BEV production. For *E. coli*, 200 ml of LB was inoculated at 1:150 and cultured for 16 h at 37 °C and 250 rpm. For LAB, 400 ml of media (MRS or CDM) was inoculated at 1:50 and cultured for 24 h at 37 °C without shaking. Then, the culture was depleted of cells and large debris by centrifugation at 10,000 g × 10 min (4 °C), followed by a second centrifugation of the supernatant at 10,000 g × 20 min (4 °C). Then, supernatant was filtered with a 0.45‐um bottle top filter (Nalgene Rapid Flow PES; Thermo #169‐0045) to generate clarified supernatant.

### 2.3 Tangential Flow Filtration (TFF)

TFF was performed using a KrosFlo KR2i TFF system (Spectrum Labs, Los Angeles, CA, USA) equipped with a 300‐kDa MWCO hollow fiber filter composed of a modified PES membrane (D02‐E100‐05‐N; Spectrum Labs). Each filter was used no more than 5 times or discarded when permeate flow rate decreased, cleaned after each use by circulating 100 mL 0.5 m NaOH for 30 min, and stored in 20% ethanol. Flow rate was set at 106 mL min^−1^ to maintain a shear rate of 4000 s^−1^ and backflow pressure was automatically adjusted to maintain a transmembrane pressure of 5 psi. Clarified supernatant was initially concentrated to 25 mL followed by diafiltration and then brought to a final concentration to ≈10 mL. Diafiltration volumes were scaled to 1.5 times the volume of clarified supernatant (600 mL for LAB and 300 mL for *E. coli*). Following TFF, samples were concentrated further to between 1–5 mL using 100 kDa ultrafiltration columns to achieve a final concentration of ≈5E11 particles mL^−1^. Finally, samples were sterile filtered with 0.2 um PES syringe filters and stored at −20 °C for no longer than 4 weeks before use.

### 2.4 BEV Characterization

Particle size and concentration were determined using NanoSight LM10 (Malvern Instruments) with Nanoparticle Tracking Analysis (NTA) software, version 2.3. Samples were diluted within the working concentration range of the NTA (particles/frame = 20–200) using PBS, and three 15‐s videos were captured with a camera level set at 14. The detection threshold was set at 3 for all samples and replicates. Total protein concentration in BEV samples was determined using Bicinchoninic acid assay (BCA; 786‐571; G‐Biosciences). BEV proteome was analyzed by 12% SDS‐PAGE followed by staining with SYPRO Ruby Protein Gel Stain (Thermo # S12001).

BEV morphology was assessed using negative stain TEM. First, 20 µL of BEV sample was mixed 1:1 with 4% EM‐grade paraformaldehyde (157‐4‐100; Electron Microscopy Sciences) for 30 min at room temperature. Throughout the following steps, ultra‐thin carbon‐coated copper grids were placed, carbon film side down, on droplets of reagents positioned on a sheet of parafilm, with excess liquid blotted between each step. First, fixed BEVs were adhered to grids by floating grids on droplet of PFA‐BEV mixture for 20 min. The BEV‐adhered grid was then washed with PBS and floated on a droplet of 1% glutaraldehyde (in ×1 PBS) for 5 min. Next, the grid was washed five times with dH20 (2‐min incubation per wash), and then negative stained using uranyl‐acetate replacement stain (22 405; Electron Microscopy Sciences) for 10 min. The grids were allowed to dry overnight before imaging on a JEOL JEM 2100 LaB6 TEM with a 200 kV accelerating voltage.

### 2.5 Macrophage Inflammatory Assay (RAW264.7, dTHP1, CCK8)

RAW264.7 mouse macrophages were seeded at a density of 75000 cells per well in a 48‐well plate with DMEM containing 10% FBS and 1% penicillin/streptomycin. After 24 h, the cells were pretreated with media supplemented with one of the following: i) ×1 PBS (six wells total), ii) BEV‐depleted conditioned media (permeate from TFF), iii) 1 µg mL^−1^ dexamethasone as a positive control (Dex; D4902‐25 MG; Sigma‐Aldrich), or iv) BEV groups isolated through different methods (TFF only, TFF + SEC, or TFF + HPAEC). All treatments were performed in triplicate, and BEV doses were normalized to particle content. After 24 h of pretreatment, the media was removed and 10 ng mL^−1^ LPS (resuspended in ×1 PBS; L4391‐1MG; Sigma‐Aldrich) was added to all wells, except for three PBS‐pretreated wells (media‐only group), to induce an inflammatory response. Conditioned media was collected 4 h after LPS stimulation, stored at −80 °C, and TNF‐α levels were measured by ELISA within three days (DY410; R&D Systems). Additionally, cell lysate was collected in QIAzol and stored at −80 C until RNA isolation within three days. For some experiments, prior to media and RNA collection, CCK8 viability assay was performed.

For human dTHP‐1, a similar process was followed as RAW264.7 with a few modifications. First, THP‐1 monocytes were differentiated into dTHP‐1 macrophages by 48 h incubation with 20 nm phorbol 12‐myristate 13‐acetate (PMA). Then, cells were pretreated with BEVs as above, followed by stimulation with 500 ng mL^−1^ LPS and 20 ng mL^−1^ IFN‐γ. Finally, 24 h after stimulation, conditioned media and/or cell lysate was collected for analysis of cytokine expression by ELISA.

### 2.6 Murine Acute DSS Colitis Model

All animal care and research were carried out using protocols approved and overseen by the University of Maryland and the University of Maryland IACUC (protocol R‐MAR‐23‐11) in compliance with local, state, and federal guidelines. Male, 8–12‐week‐old, C57BL6J mice were purchased from The Jackson Laboratory. Colitis was induced by the addition of 2.5% (w/v) dextran sulfate sodium (DSS; MW ≈40000; Chem‐Impex) to autoclaved drinking water. Mice were weighed daily, and treatments were administered by oral gavage in a 200‐ul volume. BEV doses were 2.5E9 particles/mouse/day, and live cell doses were 2.5E9 CFU/mouse/day. Sham‐treated control mice were administered vehicle (PBS). After colitis induction, DSS water was replaced with normal water constituting a “washout period”. At the end of study, colons were collected, their length measured from the ileocecal junction to rectum, and then prepared for histology by the Swiss roll method as previously described;^[^ ^38^ ^]^ mesenteric lymph nodes were collected and cells analyzed by flow cytometry; for some studies, RNA was isolated from the most proximal 1 cm of colon.

For some experiments, disease activity index was scored at the peak of disease on Day 4 and Day 5 according to the criteria in **Table** **1**, as previously described.^[^ ^39^ ^]^

**Table 1:** Disease activity index scoring criteria.

	Finding	Score
Stool consistency	Normal	0
	Loose	2
	Diarrhea	4
Blood in stool	None	0
	Trace (hemoccult positive)	2
	Grossly positive	4
Weight loss	<1%	0
	1‐5%	1
	6‐10%	2
	11‐15%	3
	>15%	4

### 2.7 Flow Cytometry

Harvested mesenteric lymph nodes (mLNs) were immediately placed on ice after collection. Then, mLNs were mechanically dissociated and passed through a 70‐um cell strainer. Cells were washed with 1x PBS + 2% FBS (FACS buffer), then blocked with anti‐CD16/CD32 antibody (TruStain FcX; clone 93; Biolegend #101 320) for 15 min at 4 °C. Following blocking, surface and LIVE/DEAD markers were stained for 30 min at 4 °C. (Zombie Near IR Fixable Viability kit; Biolegend #423 106). Then, cells were washed 2x with FACS buffer, and analyzed by flow cytometry within 12 h, or subjected to subsequent intracellular staining. For intracellular staining, cells were fixed and permeabilized using the Foxp3/Transcription factor staining buffer kit (eBioscience) and intracellular markers stained for 1 h at 4 °C. Finally, cells were washed 2x and then analyzed by flow cytometry on a FACSCelesta (BD) with data analyzed using Flowjo software (Version 9, Treestar).

### 2.8 RNA Isolation and RT‐qPCR Analysis

RNA was extracted from either mouse proximal colon tissue or RAW264.7 cell lysate. The tissue was mechanically homogenized, and the cell lysate was collected using QIAzol. Total RNA was isolated with the RNeasy mini kit (QIAGEN, 74 104, Hilden, Germany) following the manufacturer's protocol. Complementary DNA (cDNA) was synthesized from the total RNA using M‐MuLV reverse transcriptase (New England Biosciences, M0253L, Ipswich, MA, USA) as per the manufacturer's instructions. After cDNA synthesis, quantitative polymerase chain reaction (qPCR) was conducted using a QuantStudio 7 Flex qPCR system (ThermoFisher Scientific, 4 485 701, Waltham, MA, USA) and PowerTrack SYBR Green Master Mix (Thermo Scientific, A46109, Waltham, MA, USA). The primer sequences used for qPCR are provided in Table S1 (Supporting Information). mRNA transcript levels were quantified by the comparative Ct method, normalized to GAPDH expression (except in qPCR arrays), and expressed as fold changes in mRNA levels. For qPCR arrays, normalization was done using the average of four housekeeping genes (*gapdh*, *actb*, 18S rRNA, *eef1a1*). For mouse studies, RNA was isolated from individual mouse colons and converted to cDNA; then, equal masses of RNA were pooled from each mouse within a group to produce a single pooled cDNA sample for each group, RT‐qPCR was performed on the pooled sample with three technical replicates.

### 2.9 Histologic Analysis

Paraffin‐embedded Swiss rolled colons were prepared as previously described^[^ ^38^ ^]^ and sectioned into 5‐µm thick slices and stained with H&E. Stained sections of tissue were scanned into Aperio ImageScope and analyzed using methods described by Garcia‐Hernandez et al.^[^ ^40^ ^]^ Briefly, lengths of normal, inflamed, and ulcerated tissue were measured, generating a Histological Colitis Score for each sample. Ulceration was defined as complete loss of the mucosa with disruption of colonic crypt architecture, and inflammation was defined as goblet cell density loss, crypt density loss, crypt hyperplasia, and/or presence of inflammatory infiltrate or edema.^[^ ^41^, ^42^ ^]^

### 2.10 Transmission Electron Microscopy

Hypervesiculating *L. plantarum* BEV morphology was examined by transmission electron microscopy (TEM) after negative contrast staining. A 20 µL aliquot of BEVs was fixed for 30 min at room temperature in 2% EM‐grade paraformaldehyde (Electron Microscopy Sciences, 157‐4‐100). For all subsequent steps, ultrathin carbon‐supported copper grids were placed carbon side down onto small drops of reagent arranged on parafilm, with excess liquid wicked off between steps. Fixed vesicles were allowed to adsorb to the grid for 20 min, followed by a brief PBS rinse and a 5 min post‐fix in 1% glutaraldehyde prepared in 1× PBS. Grids were then washed five times in deionized water (2 min each) and negatively contrasted with uranyl‐acetate replacement stain (Electron Microscopy Sciences, 22 405) for 10 min. After air‐drying overnight, samples were imaged on a JEOL JEM‐2100 LaB6 TEM operated at 200 kV.

### 2.11 Statistical Analysis

All statistical analyses were performed using GraphPad Prism v9.0. Data from mouse studies were assessed for normality using the Shapiro‐Wilk test. Tests used for statistical significance were indicated in figure captions. Statistical significance was defined as *p* < 0.05. All data were presented as mean ± standard deviation (SD), unless otherwise specified. Sample sizes were chosen based on prior studies. No data were excluded.

## 3 Results

### 3.1 LAB BEV Characterization and Assessment of Media Effects on Production

We first characterized BEVs from selected LAB species to assess their production and physical characteristics. All four species of LAB examined here‐*L. plantarum*, *L. paracasei*, *L. reuteri*, and *L. rhamnosus* – were selected based on preliminary efficacy as single‐strain live probiotics. For example, an eight‐strain cocktail of Gram‐positive LAB (VSL#3), has shown consistent efficacy in certain human IBD subtypes (ulcerative colitis, and prevention of the post‐UC surgery complication: pouchitis). VSL#3 contains *L. plantarum and L. paracasei*, in addition to other *Lactobacillus* and *Bifidobacterium* species. BEVs from *L. plantarum* and *L. paracasei* were previously shown to be effective in mouse models of IBD.^[^ ^43^ ^]^ Additionally, *L. reuteri* has shown promising clinical efficacy as a single‐strain formulation in preventing necrotizing enterocolitis in preterm infants,^[^ ^44^, ^45^ ^]^ and also produces BEVs with anti‐inflammatory effects in animals and human PBMCs.^[^ ^46^, ^47^ ^]^ Finally, single‐strain *L. rhamnosus* was found to be equally effective as standard of care treatment for ulcerative colitis,^[^ ^48^ ^]^ and also produces BEVs with anti‐inflammatory effects in human PBMCs and efficacy in mouse models of IBD.^[^ ^46^, ^49^ ^]^

**Figure 1:**
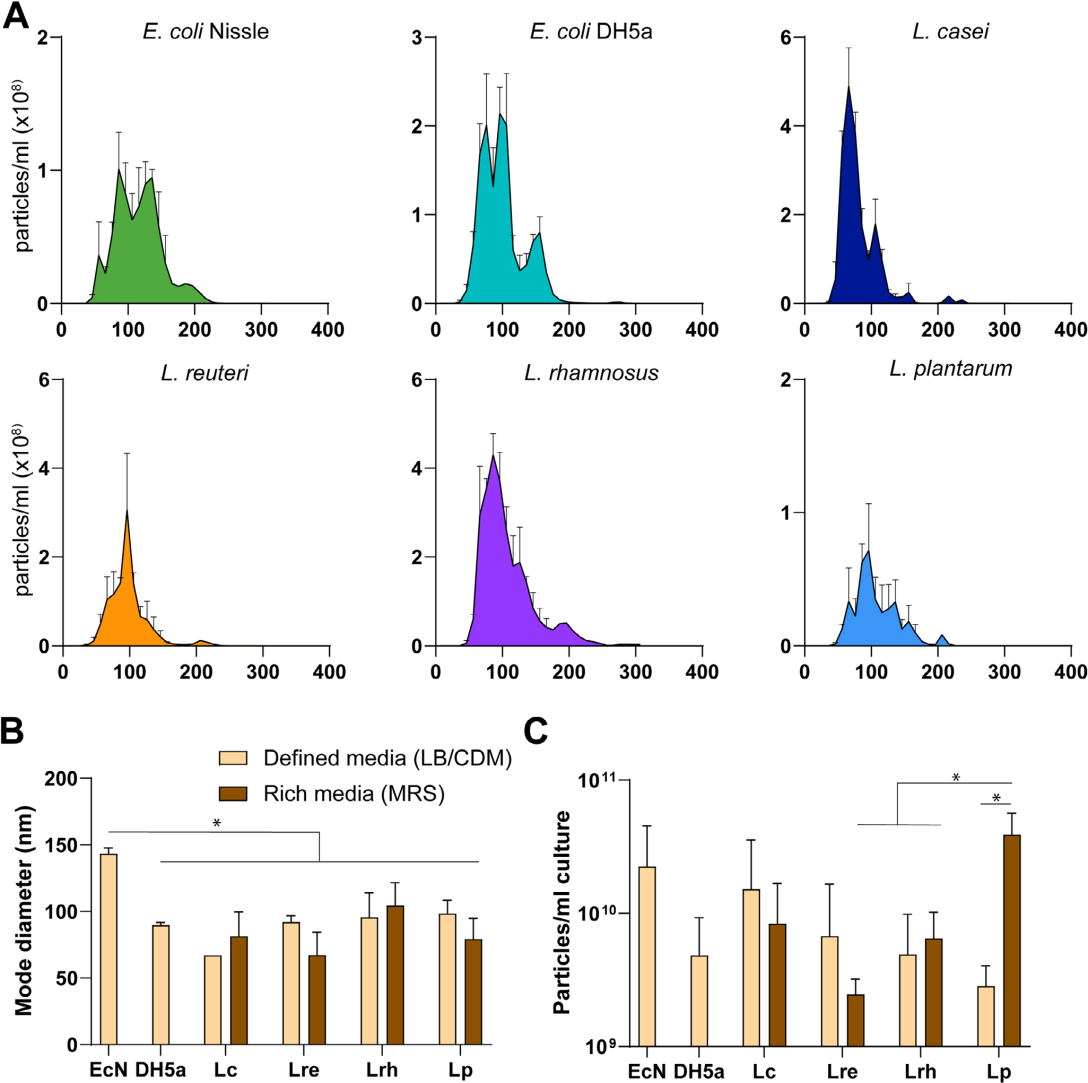
BEV size and production. A) Particle diameter distribution of BEV samples derived from different species of bacteria determined by nanoparticle tracking analysis (NTA) (error bars ± SEM), B) Mode particle diameter (nm ± SD) of BEV samples from different species of bacteria cultured in either defined media (LB or CDM) or rich media (MRS) determined by NTA, C) Total BEV yields (particles per ml culture ± SD) from flask culture determined by nanoparticle tracking analysis and normalized to volume of bacterial culture. Statistical analysis by one‐way ANOVA with Tukey post hoc test * *p* < 0.05.

LAB are typically cultivated in MRS media, a rich media‐containing animal meat extract. Digested animal tissue can contain EVs, proteins, and other large debris. Some of these products can be co‐isolated with BEVs and confound BEV proteomics and bioactivity analysis, not to mention their unsuitability for therapeutic biomanufacturing.^[^ ^50^, ^51^ ^]^ Thus, we cultivated all strains of LAB in complete defined media (CDM) that does not contain animal products, and compared BEV characteristics to standard MRS. The size distribution of particles from all species was within the expected 20–200 nm range for BEVs (**Figure** **1**A), and LAB cultured in MRS or CDM produced particles of similar size. The mode particle diameter was between 60–100 nm for all species except EcN, a probiotic *E*. *coli* species used as a control, which produced significantly larger particles with a mode size of 145 nm (Figure 1B). Of note, the other *E. coli* strain, DH5a, also produced a minor population of larger sized particles, which was insufficient to impact mode size. These findings are consistent with prior reports showing at least some Gram‐positive species produce smaller diameter particles than Gram‐negative *E. coli*.^[^ ^17^ ^]^ These smaller particles with Gram‐positive bacteria may reflect a biogenesis pathway that requires transit through the peptidoglycan cell wall. BEV yields were greater than 1E9 particles/ml culture for all species with up to ten‐fold variability in yields between species; EcN produced the highest BEV yields at ≈2E10 particles mL^−1^ culture (Figure 1C). Surprisingly, despite normal growth in CDM, *L. plantarum* produced nearly ten‐fold lower particle yields in CDM versus MRS.

### 3.2 LAB BEVs Reduce Inflammatory Responses from Mouse and Human Macrophages

Next, we aimed to apply in vitro macrophage cytokine release assays to i) confirm BEV anti‐inflammatory bioactivity, and ii) generate preliminary comparisons of anti‐inflammatory potency between different species. Here, an ideal cell source would produce BEVs with the greatest anti‐inflammatory potency, without signs of inducing toxicity in treated cells.

We screened BEV anti‐inflammatory bioactivity with well‐characterized in vitro macrophage cytokine release assays using both human and mouse cell lines. Our rationale for selecting this assay is as follows: i) IBD pathology is mediated in part by macrophage inflammatory responses, which recruits effector T cells to the intestine ultimately leading to profound inflammation and tissue damage, and ii) prior studies show macrophage inflammatory responses are regulated by LAB BEVs, potentially mediating therapeutic efficacy,^[^ ^52^ ^]^ iii) the macrophage cytokine release assay was previously shown to predict in vivo efficacy for mesenchymal stem cell derived EVs.^[^ ^53^ ^]^

**Figure 2:**
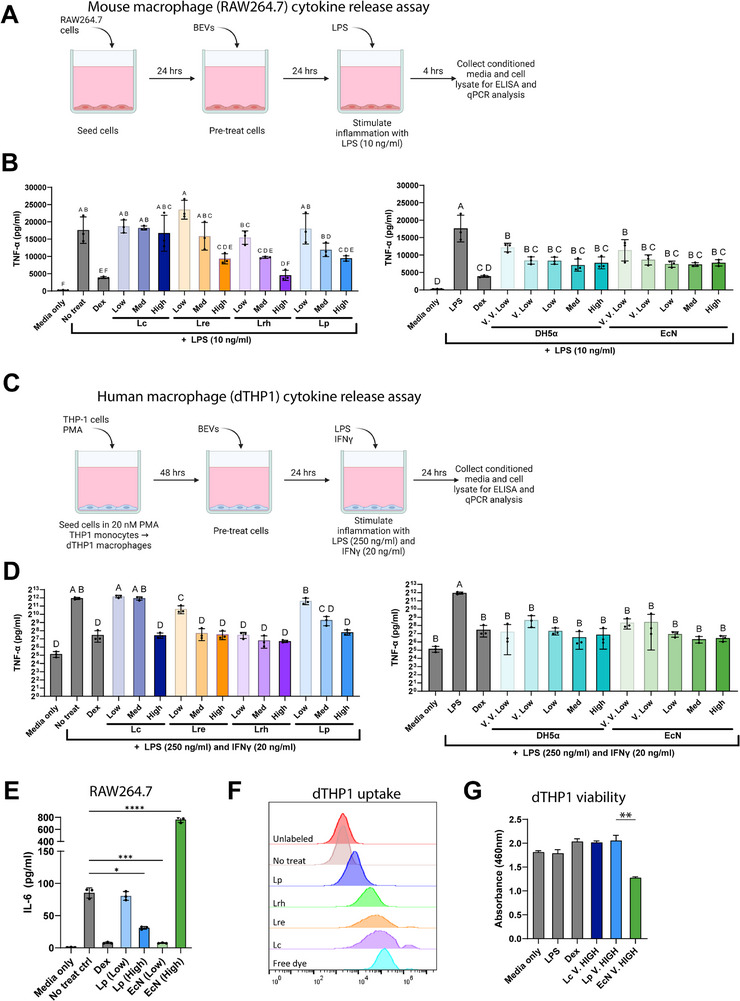
LAB BEVs exhibit anti‐inflammatory effects in vitro. A–D) Mouse RAW264.7 (A‐B) or human dTHP1 (C‐D) macrophages pretreated with BEVs display reduced inflammatory TNF‐α secretion following stimulation with 10 ng mL^−1^ LPS (for RAW264.7) or 250 ng mL^−1^ LPS + 20 ng mL^−1^ IFNγ (for dTHP1). Dosing (A–D): V.V. Low = 5E6 particles mL^−1^, V. Low = 5E7 particles mL^−1^, Low = 5E8 particles/ml, Med = 5E9 particles/ml, High = 5E10 particles mL^−1^. E) RAW264.7 macrophages pretreated with BEVs from Gram‐negative probiotic EcN BEVs or Gram‐positive probiotic *L. plantarum* (Lp) BEVs followed by LPS stimulation; Dosing (E): Low = 1E9 particles mL^−1^, High = 5E10 particles mL^−1^. F) dTHP1 macrophages were treated with 5E9 particles/ml of BEVs that were previously covalently labeled with fluorescent Alexa Fluor 647;24 h later, cell uptake of fluorescently‐labeled BEVs was analyzed by flow cytometry. Controls included BEVs not labeled with Alexa Fluor 647 (unlabeled), PBS/Vehicle treated cells (No treat), or free dye (Alexa Fluor 647 carboxylate 10 um) G) dTHP1 viability 24 h after treatment with Lc, Lp, or EcN BEVs (2e11 particles mL^−1^) and LPS+IFNγ stimulation was assessed by CCK8 assay. **Abbreviations** in figures are as follows: Lp (*L. plantarum*), Lc (*L. paracasei*), Lre (*L. reuteri*), Lrh (*L. rhamnosus*), EcN (*E. coli* Nissle 1917), DH5a (*E. coli* DH5a), Dex (Dexamathosone). Statistical significance determined with one‐way ANOVA and Tukey post hoc test. If two groups share at least one letter, their means are not significantly different (α=0.05). If they share no letters, they are significantly different (*p* < 0.05). For example, “a” versus “ab” = not significant; “a” versus “b” = significant. *p < 0.05, **p < 0.01, ***p < 0.001, ****p < 0.0001. Error bars ± SD.

BEVs from all selected LAB and *E. coli* species limited LPS‐stimulated secretion of inflammatory cytokine, TNF‐α, in mouse (**Figure** **2**B) and human macrophages (Figure 2D). However, *L. paracasei* clearly displayed lower potency in both human and mouse assays, with significant reduction in TNF‐α responses only observed at the highest dose in human dTHP‐1 macrophages (Figure 2D). Among the LAB species, *L. rhamnosus* showed potentially more potent TNF‐α suppression across both murine (Figure 2B) and human (Figure 2D) macrophages, though differences were relatively modest between BEVs from *L. rhamnoses*, *L. plantarum*, and *L. reuteri*. From these data, we conclude that *L. paracasei* BEVs show reduced in vitro anti‐inflammatory potency in LPS‐stimulated macrophages compared to BEVs from *L. rhamnosus*, *L. plantarum*, and *L. reuteri*.

With respect to *E. coli*, BEVs from probiotic (EcN) and non‐probiotic (DH5a) strains reduced TNF‐α responses at far lower (100‐fold) doses than any LAB in both human and murine macrophages (Figure 2B,D, right panel). However, at higher doses, *E. coli* BEVs stimulated IL‐6 release above levels of the no treatment control, whereas LAB BEVs reduced IL‐6 at all doses tested (Figure 2E). Among many potential explanations for these divergent responses, Gram‐negative *E. coli* BEVs contain LPS, a potent TLR4 agonist, whereas Gram‐positive LAB BEVs lack LPS but do contain TLR2 agonists (e.g., lipoproteins). Differences in bioactivity may also be explained by differences in cell uptake; however, we observed all four LAB species were taken up by macrophages without correlation between uptake (efficiency or MFI) and reduction of TNF‐α responses from macrophages (Figure 2F). Of note, BEVs are taken up by multiple pathways and we did not analyze specific uptake pathways.^[^ ^54^ ^]^ Finally, a key difference between LAB and *E. coli* BEVs was effects on cell viability at high doses (Figure 2G). Since LAB BEVs naturally lack LPS (endotoxin) which can cause toxicity, we hypothesized LAB BEVs would preserve cell viability at high doses. Indeed, LAB BEVs at high doses, up to 2e11 particles mL^−1^ (maximum feasible dose), did not reduce cell viability. On the other hand, *E. coli* (EcN) BEVs at high doses reduced cell viability.

### 3.3 LAB BEVs Show Efficacy in Acute DSS Colitis Murine Model

For a more definitive comparison of therapeutic efficacy of BEVs from different LAB species, we evaluated candidate BEVs in a well characterized preclinical mouse model of IBD (ulcerative colitis) – acute dextran sulfate sodium (DSS) induced colitis.^[^ ^55^ ^]^ In the DSS colitis model, DSS ingested from drinking water forms nanoliposomal complexes in the intestines that are toxic to intestinal epithelial cells. Death of intestinal epithelial cells compromises barrier integrity, leading to increased flux of bacterial antigens from the microbiome into host tissues, triggering a massive inflammatory response. Ultimately, moderate to severe colitis develops which can be assessed by several endpoints including i) colitis‐induced colon shortening, ii) disease activity index (DAI; weight loss, diarrhea, bleeding), iii) histology, and iv) cell populations and gene expression. For this screen, we focused on colitis‐induced colon shortening owing to its widespread use to evaluate treatment efficacy in acute DSS murine models and its amenability to high throughput analysis. We supplemented this measurement by analyzing T cell populations in the mesenteric lymph nodes and colon tissue gene expression.

**Figure 3:**
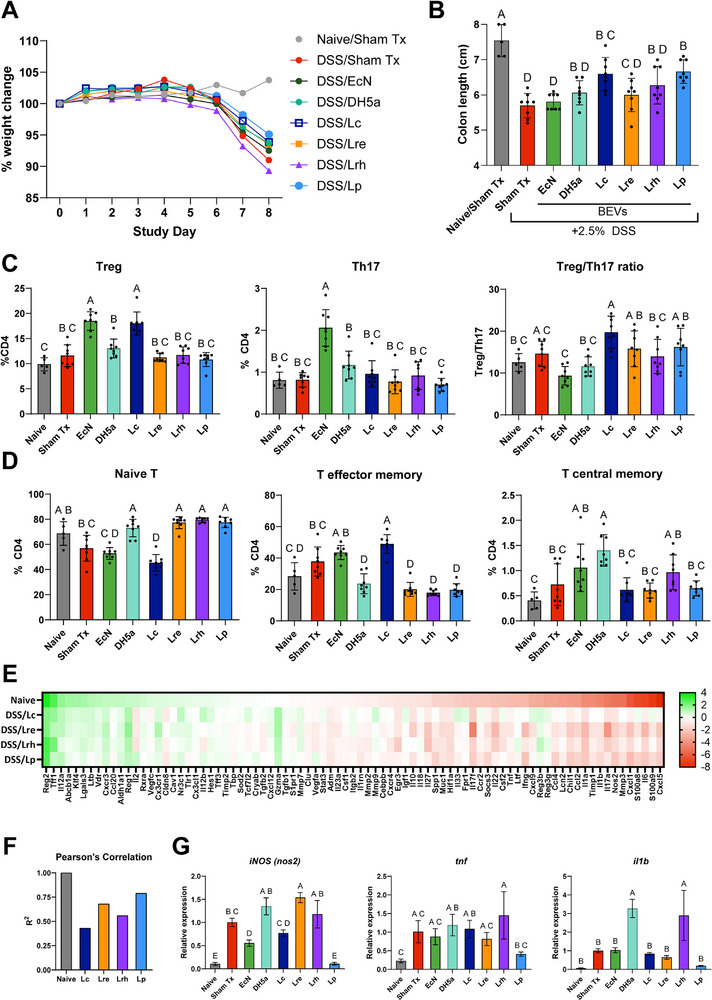
LAB BEVs reduce severity in acute DSS‐induced colitis. Mice received BEVs (2.5E9 particles mouse^−1^ day^−1^, oral gavage, Days 1–7). DSS (2.5%) was given in drinking water Days 0–6, then normal water Days 7–8 (washout). A) Body‐weight change relative to Day 0. B) Colon length at Day 8. C,D) Mesenteric lymph‐node CD4+ T‐cell populations/activation at endpoint: Treg (CD4+CD8−CD25+Foxp3+), Th17 (CD4+CD8−Foxp3−RORγt+), Naive (CD4+CD44−CD62L+), Effector (CD4+CD44+CD62L−), Central memory (CD4+CD44+CD62L+). E) qPCR array of human UC–associated genes in proximal colon, shown as log2 fold‐change versus sham (vehicle/PBS) colitis. F) Pearson correlation of colitis‐associated genes comparing treatment groups with naïve/healthy mice; R^2 closer to 1 indicates greater similarity to naive. G) RT‐qPCR of selected colitis genes in pooled, bulk colon RNA. Abbreviations: *L. plantarum* (Lp), *L. paracasei* (Lc), *L. reuteri* (Lre), *L. rhamnosus* (Lrh), *E. coli* Nissle 1917 (EcN), *E. coli* DH5a (DH5a). Statistics: one‐way ANOVA with Tukey post hoc; different letters *p* < 0.05. If two groups share at least one letter, their means are not significantly different (α = 0.05). If they share no letters, they are significantly different (*p* < 0.05). For example, “a” versus “ab” = not significant; “a” versus “b” = significant.

Following treatment, we found that BEVs from *L. plantarum* and *L. paracasei* significantly reduced colitis‐induced colon shortening. Trends for reduced weight loss were also observed for these BEV treatment groups, but differences did not reach statistical significance (**Figure** **3**A,B). Additionally, *L. plantarum* and *L. paracasei* BEVs reduced colitis‐induced colon shortening significantly more than probiotic *E. coli* (EcN) BEVs, which had no significant effect on colon length at the 2.5E9 particles/mouse/day dose. Furthermore, *L. plantarum* and *L. paracasei* BEVs showed non‐significant trends for greater reductions in colitis‐induced colon shortening compared to other LAB BEVs (*L. reuteri* and *L. rhamnosus*).

To explore potential mechanisms of BEVs from different LAB species and further validate conclusions of efficacy beyond colon length measurements, we evaluated T cell populations in mesenteric lymph nodes and gene expression in bulk colon tissue. T cells are critical drivers of human IBD, with therapeutic relevance established most notably by the monoclonal antibody vedoluzimab (anti‐α4β7), which inhibits T cell homing to intestinal tissues to reduce intestinal inflammation and tissue damage. In a 7 day acute DSS model, early T cell activation and polarization can be observed by Day 7; however, peak T cell clonal expansion and polarization is expected to occur later in Days 10–14. While BEVs are not reported to be taken up by T cells to a significant degree, they may indirectly signal to T cells via antigen presenting cells, warranting investigation of T cell responses.

Between naive and sham treatment control groups, we found no significant differences in T cell populations (Figure 3C) or T cell activation (Figure 3D), consistent with the relatively short 7‐day disease model. We observed no significant differences in Treg and Th17 populations between all LAB BEV treatments and sham treatment, except *L. paracasei* BEVs that significantly increased Treg populations by ≈50% without increases in Th17 populations (Figure 3C). In contrast, EcN BEVs significantly increased both Th17 and Treg populations, highlighting an important divergent T cell mechanism between LAB and EcN BEVs. We did observe increased T cell activation (reduced naive, increased effector) with *L. paracasei* and EcN BEV treatment, consistent with their expansion of Treg and Treg+Th17 populations, repectively. Furthermore, several LAB BEV groups showed slighly reduced T cell activation versus sham treatment, including *L. plantarum* BEVs. In summary, we found divergent T cell responses between LAB BEVs (especially *L. paracasei)* and EcN BEVs, with LAB BEVs showing potential greater regulatory bias versus EcN BEVs. We also found preliminary evidence that some LAB BEVs may reduce T cell activation in colitis; however, the study was not desiged to investigate T cell activation and this observation warrents further investigation as a potential mechanism of action of LAB BEVs in murine colitis treatment.

Next, we analyzed gene expression changes in bulk colon tissue using a qPCR array of genes linked to human ulcerative colitis. As expected, DSS‐induced colitis was associated with increased pro‐inflammatory genes (*il1b, tnf, ifng*, and *nos2*), elevated surrogate markers of disease severity (*s100A8*, *s100A9*, and *lcn2*), and reduced expression of colitis‐protective genes (*reg1*, *reg2*, *tff1*, *vdr*, and *abcb1a*) (Figure 3E). Collectively, gene expression changes between naive and colitis mice defined a “colitis‐associated gene expression profile”. Consistent with colon length data, global analysis of gene expression data using Pearson's correlation analysis revealed *L. plantarum* BEVs best normalized the colitis‐associated gene expression profile (R^2^ = 0.79, correlated to naive) (Figure 3F). Surprisingly, *L. reuteri* BEVs showed the 2^nd^ strongest correlation to naïve mice (R^2^ = 0.68), despite no significant effect on colon length. Finally, *L. plantarum* BEV treatment reduced key inflammatory markers to a greater extent than other BEV treatments (Figure 3G).

In summary, we identified *L. plantarum* as a suitable candidate cell source for continued development of BEVs based on improvements in colitis‐induced colon shortening and global gene expression with accompanying reduced weight loss versus BEVs from other cell sources.

### 3.4 Validation of *L. plantarum* BEV Efficacy in Acute DSS Colitis Model

To further validate the potential of *L. plantarum* BEVs for IBD therapy, we used the acute DSS mouse model as before, with the following treatment groups: i) two doses of *L. plantarum* BEVs (2.5E9 and 1E10 particles/mouse/day) and ii) a commercially available probiotic formulation previously shown effective in small randomized controlled trials (VSL#3), iii) single strain live *L. plantarum* cells, and iv) FDA‐approved 1^st^ line drug for ulcerative colitis (5‐ASA). These treatments were selected due to their predominant use in patients with ulcerative colitis – a likely initial application of therapeutic BEVs.^[^ ^16^ ^]^ The lower dose of BEVs (2.5E9 particles/mouse/day) was the same as prior studies, the higher dose (1E10 particles/mouse/day) was selected to assess efficacy and safety at supratherapeutic doses and guide determination of a therapeutic window.

**Figure 4:**
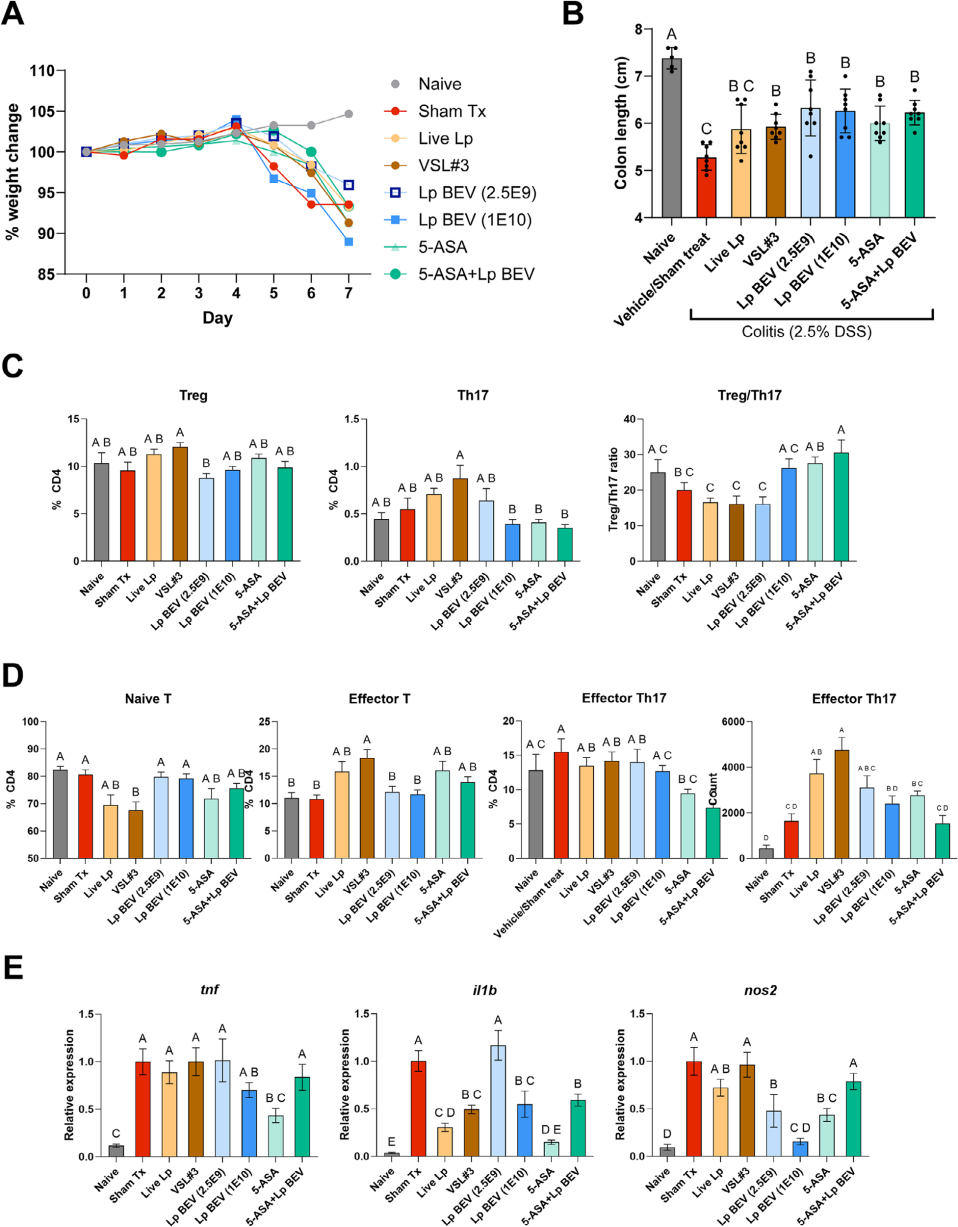
*L. plantarum* BEVs reduce murine colitis severity with potentially more favorable immunomodulatory profile compared to live probiotic cells. Mice received 2.5% DSS (drinking water, Days 0–5) then water (Days 6–7). Treatments were given by oral gavage daily (Days 1–7): live *L. plantarum* (2.5E9 CFU mouse^−1^ day^−1^), VSL#3 (2.5E9 CFU mouse^−1^ day^−1^), 5‐ASA (200 mg kg^−1^ day^−1^), or *L. plantarum* BEVs + 5‐ASA (BEVs 2.5E9 particles mouse^−1^ day^−1^). *N *= 8 per group (naïve *n* = 5). A) Body‐weight change (% baseline). B) Colon length at Day 7. C,D) Live single CD4+ T‐cell populations in mesenteric lymph nodes (means ± SEM): Treg (CD25+FOXP3+), Th17 (CD25+FOXP3−RORγt+), Naïve T (CD44−CD62L+), Effector T (CD44+CD62L−), Effector Th17 (CD44+CD62L−RORγt+). E) RT‐qPCR of bulk colon tissue normalized to GAPDH; equal cDNA mass per mouse pooled; 3 technical replicates. Statistics: one‐way ANOVA with Tukey post hoc. If two groups share at least one letter, their means are not significantly different (α = 0.05). If they share no letters, they are significantly different (p<0.05). For example, “a” versus “ab” = not significant; “a” versus “b” = significant.

As before, we found mice lost ≈5–10% body weight during colitis onset, without significant differences in weight loss across treatment (**Figure** **4**A). Differences in colitis‐induced colon shortening were significant, with all treatment groups except live *L. plantarum* cells showing significantly reduced colon shortening, indicative of reduced colonic tissue damage and therapeutic efficacy (Figure 4B). Interestingly, *L. plantarum* BEVs showed a non‐significant trend for superior improvements in colon length compared to both live probiotic groups (Live *L. plantarum* cells and VSL#3) and 5‐ASA. Finally, we included a co‐treatment group of *L. plantarum* BEVs (2.5E9 particles mouse^−1^ day^−1^) with 5‐ASA (200 mg kg^−1^ day^−1^) and found reductions in colitis‐induced colon shortening with this combination, and trends for greater reductions compared to either treatment alone, suggestive of synergistic efficacy.

Next, we evaluated T cell populations in mesenteric lymph nodes. Here, as in prior studies, *L. plantarum* BEVs did not polarize T cells toward a regulatory phenotype (Figure 4C). *L. plantarum* BEV treatment at 1E10 particles mouse^−1^ day^−1^ did significantly reduce populations of Th17 inflammatory cells. Likewise, 5‐ASA and 5‐ASA + *L. plantarum* BEV treatments significantly reduced Th17 populations. In contrast, live probiotics (*L. plantarum* cells or VSL#3) did not reduce Th17 populations, and in fact showed an increase in the mean percentage of Th17 cells.

We again assessed the activation of T cells using CD44 and CD62L markers as in Figure 3. *L. plantarum* BEVs did not increase T cell activation like prior studies. We found essentially no differences in T cell activation in naive and sham Tx groups, and no difference in T cell activation between *L. plantarum* BEVs and Sham treat. Most interestingly, VSL#3 live probiotics significantly increased activated effector T cell populations, and a similar trend that did not reach statistical significance for live *L. plantarum* cells (Figure 4D). In contrast, *L. plantarum* BEVs showed significantly reduced T cell activation compared to VSL#3. Altogether, the data support generation of a hypothesis that *L. plantarum* BEVs have a more favorable impact on T cell responses compared to live probiotics during murine colitis; this may be due to potency differences, presence of immunostimulatory compounds in probiotic cell formulations, or bona fide distinct mechanisms of action of *L. plantarum* BEVs.

Finally, we assessed expression of key inflammatory markers in the colon tissue of mice: i) *tnf*, an inflammatory cytokine target in IBD treatment, ii) *nos2*, an inflammation‐induced producer of reactive nitrogen species that contribute to oxidative tissue damage, and iii) *il1b*, a potent inflammatory cytokine (Figure 4E). All these genes are primarily expressed by intestinal epithelial cells and myeloid immune cells that could take up BEVs and are implicated in IBD pathogenesis. We found *L. plantarum* BEVs reduced *nos2* expression significantly greater than both live probiotic treatments, and these effects were meaningful; live probiotics showed no reduction in *nos2* expression whereas *L. plantarum* BEVs showed dose‐dependent reductions in *nos2* up to 90% below sham‐treated mice. This marker *nos2* bears clinical significance as a mediator of inflammation‐induced tissue damage and warrants further investigation as a distinguishing mechanism between live probiotics and their secreted BEVs.

From these studies, we conclude that: 1) *L. plantarum* BEVs show comparable preclinical efficacy in acute DSS murine models of IBD to products commonly used in management of ulcerative colitis (VSL#3 and 5‐ASA); 2) *L. plantarum* BEV treatment is potentially compatible in combination with current standard‐of‐care first‐line therapy in mild to moderate ulcerative colitis (5‐ASA).

### 3.5 Generation of a Hypervesiculating Strain of *L. plantarum* for Therapeutic BEV Production

**Figure 5:**
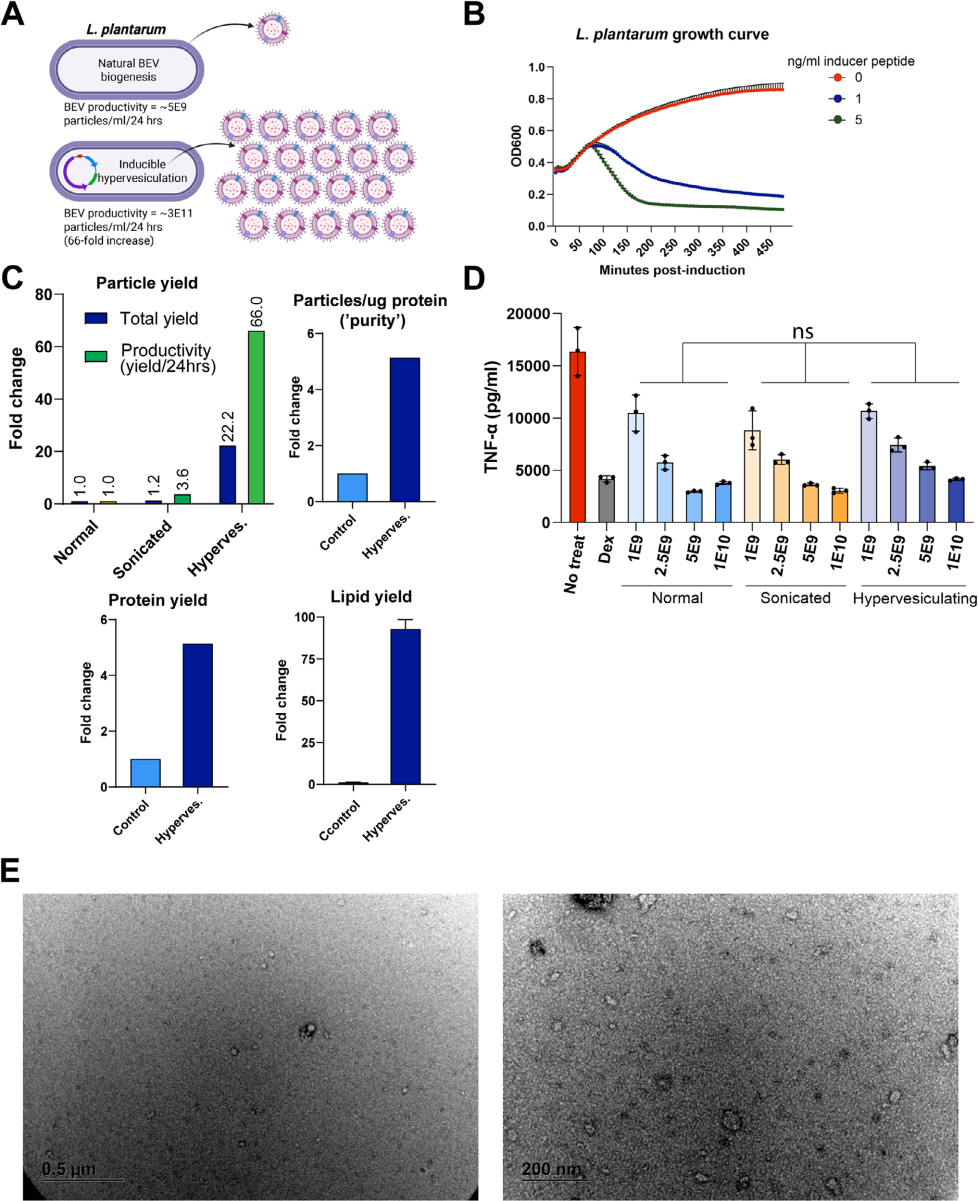
Engineering and characterization of hypervesiculating *L. plantarum* BEVs. A) Design of a genetically programmed hypervesiculating *L. plantarum* with inducible activation. B) Growth kinetics: cultures in 96‐well plates (starting OD600≈0.4) with sppIP inducer peptide at 0, 1, or 5 ng mL^−1^; OD600 monitored over time. C) BEV yields from 100‐mL flasks: wild‐type (Ctrl) cultured 24 h; hypervesiculating strain cultured ≈4–6 h, induced (100 ng mL^−1^ inducer peptide), and harvested 2 h post‐induction; sonicated cell pellet. BEVs quantified by nanoparticle tracking analysis (particles), bicinchoninic acid assay (protein), and FM4‐64 dye (lipid); values not normalized to cell count or OD600. Purity calculated as particles µg^−1^ total protein. The sonicated control was prepared from an equal‐volume cell pellet collected at 8 h OD_600_ = 1 (resuspended in 30 mL PBS; 3 × 30‐s pulses) followed by BEV isolation. D) RAW264.7 macrophages pretreated with BEVs then stimulated with LPS show no significant differences in TNF‐α secretion among normal BEVs, sonicated‐pellet BEVs, and hypervesiculating BEVs (two‐way ANOVA; *n* = 3 technical replicates), E) Electron micrographs of hypervesiculating *L. plantarum* BEVs prepared via negative staining method.

Upon confirmation that *L. plantarum* is a desirable cell source for production of therapeutic BEVs, we moved to solve the critical problem of low BEV production yields. Our approach was based on the premise that bacteria possess exceptional capacity for genetic engineering to augment or introduce desirable functions, and this can be applied for large scale increases in BEV yields. Thus, our goal was to genetically program *L. plantarum* to mass produce BEVs (“hypervesiculating” strain) with validated IBD therapeutic potential. Our design strategy targeted a natural mechanism of BEV biogenesis, modulation of peptidoglycan cross‐linking, by inducible expression of a peptidoglycan remodeling enzyme using pSIP403 (**Figure** **5**A).^[^ ^56^ ^]^ We found this genetic circuit increased BEV production rates >66‐fold with 22‐fold increased total BEV yields in just 8 h compared to conventional 24‐h culture (based on particle counts) (Figure 5B,C). An orthogonal method of BEV yield quantification (lipid quantification) showed even greater yield increases (Figure 5C). Further, not only were BEV yields dramatically increased, but also BEV purity dramatically increased based on an established BEV purity metric: particles per ug protein (Figure 5C).^[^ ^57^, ^58^ ^]^ We note that under some experimental conditions BEV yield increases were associated with concurrent reductions in cell density measured by OD_600_, but in other experimental conditions increased BEV yields were not associated with reduction in cell density; these conditions were distinguished by varying OD_600_ at induction, dose of inducing peptide, and timing of BEV isolation following induction (Figure S1, Supporting Information). Therefore, we reason that complete cell lysis is not required for hypervesiculation.

Finally, hypervesiculating *L. plantarum* BEVs showed in vitro anti‐inflammatory effects in an LPS‐stimulated mouse macrophage cytokine release assay (Figure 5D) and displayed typical morphology as determined by transmission electron microscopy (Figure 5E). From these data, we concluded that hypervesiculating *L. plantarum* BEVs were suitable for continued validation.

### 3.6 Hypervesiculating *L. plantarum* BEVs are Effective in an Acute DSS Murine Model of IBD

**Figure 6:**
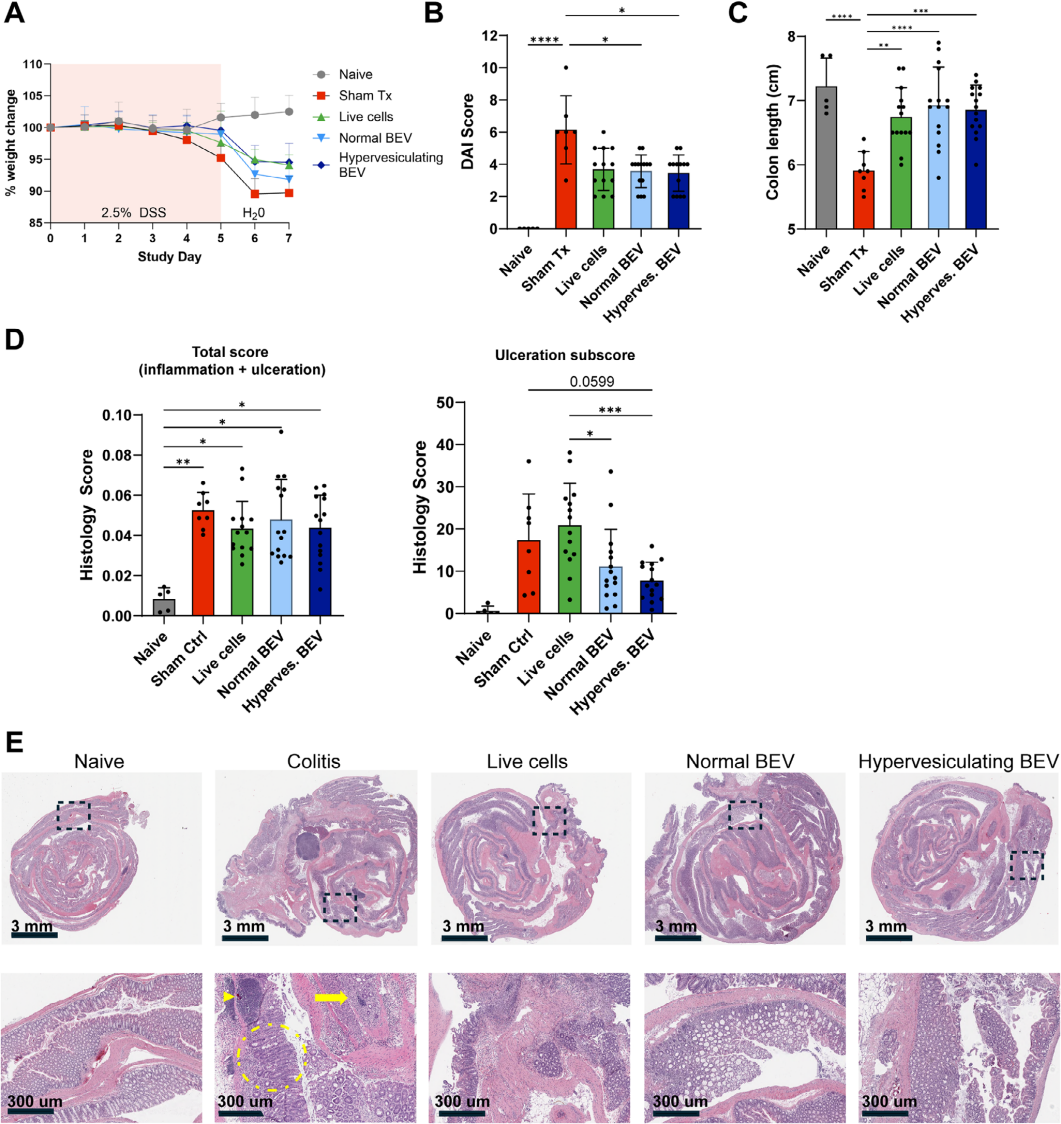
Hypervesiculating *L. plantarum* BEVs are effective in murine model of acute DSS‐induced colitis. Mice undergoing acute DSS‐induced colitis were treated with hypervesiculating *L. plantarum* BEVs (Hyperves. BEV), normal BEVs from *L. plantarum*, live *L. plantarum* cells, or vehicle (saline (PBS); Sham Tx) daily from Day 1 – Day 6 via oral gavage, and 2.5% DSS was administered in drinking water from Day 0‐Day 5. BEV doses were 2.5E9 particles/mouse/day, and the live cell dose was 2.5E9 CFU mouse^−1^ day^−1^. *N* = 15 for treatment groups, *N* = 8 for Sham ctrl, *N* = 4 for naive. A) Body weight changes of mice during acute DSS colitis relative to Day 0 weight, B) Colon length at study endpoint (Day 7), C) Disease activity index (DAI) was assessed on Day 5. D) Mean Histological Colitis Scores and ulceration subscores derived from mouse colon sections. Histological analysis of H&E‐stained colon sections was performed by a blinded pathology team to assess tissue inflammation and tissue damage (ulceration). E) Representative images depict hypervesiculating *L. plantarum* BEVs reducing tissue damage relative to control mice treated with saline and live cell‐treated mice. Dashed boxes indicate areas magnified below each image. Arrowhead indicates crypt abscess; arrow indicates ulcer; dashed circle indicates area of crypt hyperplasia and inflammatory infiltrate. Panels B‐D show means and standard deviation; statistical significance was determined by one‐ or two‐way ANOVA and Tukey post hoc test. *p < 0.05, **p < 0.01 ***p < 0.001, ****p < 0.0001.

Finally, we sought to evaluate hypervesiculating *L. plantarum* BEV efficacy in the acute DSS colitis model. Mice were orally administered one of three treatments: i) live *L. plantarum* cells, ii) normal *L. plantarum* BEVs, iii) hypervesiculating *L. plantarum* BEVs; control mice were administered saline (sham treat). A reliable and accurate dose normalization method between cells and EVs is not possible, so we elected to use a dose of cells at the upper range of literature reports to allow preliminary comparisons of treatment efficacy and mechanisms.^[^ ^59^, ^60^, ^61^, ^62^ ^]^ Over the course of the study, all treated mice showed a trend for reduced weight loss compared to sham control, with ≈10% weight loss at Day 7 for sham versus ≈5% for all three treatment groups (ns) (**Figure** **6**A). We also found significantly reduced disease activity score (DAI) at the peak of disease (Day 5) for normal and hypervesiculating *L. plantarum* BEVs (Figure 6B). At the end of the study, we found significantly reduced colitis‐induced colon shortening for all treatment groups (Figure 6C). There was no significant difference in colon length between the three treatment groups. Finally, blinded analysis of H&E‐stained colon sections revealed reduced tissue ulceration in hypervesiculating *L. plantarum* BEV‐treated mice versus sham (*p* = 0.0599); whereas live *L. plantarum* cell‐treated mice showed no improvement (Figure 6D). Hypervesiculating *L. plantarum* BEVs also showed a trend for greater reductions in tissue ulceration compared to normal *L. plantarum* BEVs.

## 4 Discussion

BEVs offer intriguing therapeutic potential for IBD, combining oral bioavailability and a predictable pharmacokinetic/pharmacodynamic profile with the capability of being engineered for versatile drug delivery. However, current production yields of BEVs are low and limit the potential for clinical translation. Thus, we initially sought to identify a suitable bacterial strain in which to implement genetic engineering solutions to low BEV production yields while capitalizing on inherent therapeutic potential for IBD. We used a combined rational and empirical approach, identifying genetically tractable cell sources with promising early clinical efficacy and safety as live cell formulations and then experimentally evaluating therapeutic activity of isolated BEVs. Strengths of our approach include experimental design based on interventional clinical trial data and inclusion of both Gram‐positive LAB species and Gram‐negative *E. coli* species. Probiotic *E. coli* Nissle 1917 BEVs are under development as vaccines and have reported efficacy in animal colitis models. This comparison is key, since technologies to increase BEV yields and load EcN BEVs with IBD therapeutic protein cargo have already been developed. Live EcN has shown efficacy in human IBD equivalent to standard of care therapy,^[^ ^63^ ^]^ and EcN BEVs have efficacy in mouse models of IBD.^[^ ^64^ ^]^ Thus, *E. coli* BEV's technology portfolio could support development of EcN BEV therapeutics for IBD. Limitations of our approach include constraints on the number of cell source candidates due to lack of validated high throughput potency assays and low throughput of animal disease models. Additionally, we utilized the type strain of each LAB species and note that strain‐specific differences could result in altered BEV bioactivity.

To collect empirical data of BEV therapeutic activity, we employed a combination of in vitro and in vivo bioactivity screens. For in‐vitro activity, we used an LPS‐stimulated macrophage cytokine‐release assay to assess immunomodulation and viability, which has previously been used to assay LAB immunomodulatory activity. Macrophages readily internalized LAB BEVs, which dose‐dependently reduced the secretion of the inflammatory cytokine, TNF‐α, following LPS stimulation (Figure 2). TNF‐α is a critical cytokine driving colitis and is a therapeutic target in human IBD (UC and CD). We note the macrophage assay uses a single early time point and therefore provides a snapshot rather than a kinetic profile, and in vitro studies relied on a single cell type (mouse and human macrophages). Future work will expand to later time points (e.g., IL‐10 kinetics) and intestinal epithelial assays to evaluate barrier and epithelial inflammatory endpoints.

EcN BEVs suppressed TNF‐α at ≈100‐fold lower doses than LAB BEVs (Figure 2), but at higher doses drove marked IL‐6 induction—exceeding purified LPS—and reduced cell viability, likely reflecting LPS content. On the other hand, LAB BEVs suppressed IL‐6 at all doses tested without reductions in cell viability. Like TNF‐α, IL‐6 plays an important role in pathogenesis of chronic inflammatory and autoimmune diseases, and is a therapeutic target in rheumatoid arthritis, but not IBD. Thus, EcN induction of IL‐6 responses from macrophages may detract from therapeutic efficacy in chronic inflammatory diseases with unclear relevance to IBD. Several genetic strategies exist to detoxify LPS present on EcN BEVs, which could alter EcN BEV's effects on IL‐6 and in vitro cell toxicity.

In the acute DSS murine colitis model, both *L. plantarum* and *L. paracasei* BEVs significantly reduced disease severity measured by colitis‐induced colon shortening, and *L. plantarum* and *L. reuteri* BEVs most effectively normalized the colitis‐associated gene expression profile measured by Pearson's correlation analysis (Figure 3). *L. plantarum* BEV efficacy in acute DSS was validated over multiple experiments and benchmarked against a FDA‐approved IBD therapeutic (5‐ASA). Thus, we concluded that *L. plantarum* BEVs were a suitable cell source to apply engineering to address low BEV production yields.

We prioritized the acute DSS model for the studies herein due to throughput and its modeling of epithelial injury and innate immune activation pathways known to be targeted by LAB BEVs. Future efficacy validation in other animal models, including chronic disease models (IL‐10 knockout, adoptive T cell transfer, and/or anti‐CD40 colitis), will be useful to establishing dosing, mechanisms, and further support clinical development. Additionally, only male mice were used, due to developing more severe disease than females, but sex‐specific difference in IBD warrant studies in female mice. Finally, we pooled equal RNA mass within groups to expand gene coverage at the expense of masking biologic variability; to address this limitation, we performed per‐mouse confirmatory qPCR on key transcripts (*Tnf, Il1b, Nos2, S100a8*, and *S100a9*) in key treatment groups (naïve, sham, and *L. plantarum* BEV), with per‐mouse expression consistent with pooled‐RNA findings and showed significant decreases (Figure S2, Supporting Information).

Prior to the study, we anticipated BEVs would show similar mechanisms of action as their producer cell (live probiotics), but potentially higher potency. Instead, we observed a marked divergence across oxidative‐stress signaling, T cell polarization, and mucosal injury in acute DSS colitis (Figure 4). *L. plantarum* BEVs dose‐dependently suppressed nos2 (iNOS) in colon tissue—by up to ≈90% across studies—whereas live *L. plantarum* and a multi‐strain LAB probiotic containing *L. plantarum* (VSL#3) showed no change (Figure 4E). Consistent with this, *L. plantarum* BEVs reduced Th17 populations, while live probiotics (VSL#3 and live *L. plantarum)* did not. In fact, VSL#3 significantly increased activated effector T cells, and VSL#3 and live *L. plantarum* showed non‐significant trends for reduced Treg:Th17 ratio and increased Th17 populations (Figure 4C,D). Finally, and most critically for therapeutic outcomes, BEVs significantly reduced mucosal ulceration—an endpoint aligned with clinical IBD trials—whereas live *L. plantarum* showed no improvement and even a marginal worsening in mean ulceration score compared to sham treat control. Together, these data suggest either dramatically higher potency of BEVs and/or distinct mechanisms of action for BEVs relative to their producer cells. Potential distinct mechanisms of action warrant further investigation, such as in dendritic cell‐T cell co‐culture assays, longer duration animal studies, and T cell‐dependent mouse models of colitis (e.g., TNBS, adoptive T cell transfer), as well as identifying the specific components of BEVs responsible for our observed bioactivity. With respect to BEV composition, BEVs contain certain cargo enriched relative to producer cells, and other cargo depleted relative to producer cells; distinct BEV cargo creates possibility for distinct mechanisms. For example, *L. plantarum* can produce the phospholipid‐derivative lysophosphatidic acid (LPA), which can promote inflammation in a variety of chronic inflammatory diseases^[^ ^65^ ^]^ and is significantly lower in abundance in *L. plantarum* BEVs relative to producer cells.^[^ ^66^ ^]^ In another example, *L. plantarum* synthesizes the glycolipid, GL1 (α‐glucosyl diacylglycerol), which is a ligand for the C‐type lectin receptor, Mincle.^[^ ^67^ ^]^ While Mincle engagement alone can promote inflammatory responses, co‐engagement of Mincle and TLR2 synergizes to increase anti‐inflammatory IL‐10 expression from dendritic cells.^[^ ^68^ ^]^

Conceptually, BEVs carry cargo from their producer cell, suggesting overlap with live bacteria mechanisms. However, specific classes—lipids, lipoproteins, peptides, and RNA—can be enriched in BEVs and delivered more efficiently when BEV‐encapsulated. For example, Gram‐positive bacterial lipoproteins are established TLR2 agonists, and recent work shows their regulated export within BEVs, clarifying a secretion route. Indeed, wild type *L. plantarum* BEVs contain lipoproteins and lipoteichoic acid that can signal through TLR2/1 and TLR2/6 heterodimers; we confirmed hypervesiculating *L. plantarum* BEVs also engage TLR2 signaling using TLR2 reporter cells (Figure S3, Supporting Information). TLR2 signaling protects from colitis,^[^ ^69^ ^]^ TLR2 mutations in humans correlate with increased IBD severity, and TLR2 agonists can treat murine colitis^[^ ^69^, ^70^ ^]^ but present risks of systemic toxicity. TLR2 is highly expressed in intestinal epithelial cells and myeloid immune cells – key cellular targets of BEV delivery – and engagement of TLR2 promotes innate immune tolerance and increased mucosal barrier integrity, consistent with our data.

Beyond TLR2, *L. plantarum* expresses small RNAs that are enriched in *L. plantarum* BEVs; these small RNA sequences, when synthesized, purified, and applied to human cells in vitro, effectively inhibit expression of genes linked to inflammation, suggesting BEV‐associated small RNA could regulate human gene expression and improve colitis outcomes.^[^ ^71^ ^]^ However, direct evidence of BEV‐associated small RNA regulating mammalian gene expression has yet to be demonstrated. BEV‐mediated delivery is a critical aspect to this potential mechanism, since RNA is extremely labile in the GI tract, but BEVs have shown stability in GI fluids,^[^ ^72^ ^]^ which we hypothesize is due to their distinct lipid composition, potentially evolved to mediate delivery of bacterial‐derived compounds to human tissues in the GI environment. Another class of molecules expressed by *L. plantarum* and present in BEVs are long‐chain lipids, which require solubilization in aqueous environments like the GI tract for delivery to human tissues, which may be enabled by solubilization in the BEV lipid membrane hydrophobic core. Standard discovery screens that use filtered (± concentrated) supernatants favor abundant, soluble, membrane‐permeant metabolites (e.g., SCFAs), potentially under‐detecting vesicle‐delivered effectors. We therefore view BEVs as a distinct bacterial secretion system that can traffic lipid‐soluble molecules and/or otherwise unstable biologics (peptides, proteins, RNA) from the GI lumen to host tissues. Additionally, we note a limitation of our study is lack of detail on the specific LAB BEV cargo driving therapeutic efficacy. Elucidation of such cargo will be critical in establishing mechanisms of how *L. plantarum* BEVs reduce murine colitis severity and improve inflammatory markers, T cell responses, and mucosal tissue ulceration. This knowledge can inform clinical trial design, potency assay development, and identification of novel therapeutic molecules within BEVs. *L. plantarum* BEVs are known to contain abundant TLR2 ligands (lipoproteins, lipoteichoic acid), and TLR2 engagement is known to drive innate immune modulation and reduced mucosal ulceration in murine colitis. *L. plantarum* BEVs also contain additional bioactive molecules potentially contributing to therapeutic efficacy. For example, recent studies reveal *L. plantarum* expresses potent peptide agonists of formyl peptide receptor 2 (FPR2),^[^ ^73^ ^]^ a G‐protein coupled receptor (GPCR) linked to resolution of inflammation in colitis, and *L. plantarum* BEVs activate FPR2 signaling in FPR2 reporter cells.^[^ ^74^ ^]^ The presence of FPR2 agonists in *L. plantarum* BEVs has yet to be confirmed.

Any BEV yield‐generating method must generate order‐of‐magnitude increases in BEV yields to meaningfully improve BEV biomanufacturing. Previous approaches in Gram‐positive bacteria have included: i) cell disruption (e.g., French press)^[^ ^75^ ^]^ or ii) environmental modification (e.g., pH, temperature, media agitation).^[^ ^19^ ^]^ Unfortunately, these approaches produce at best only 2‐5‐fold increased BEV yields and are not scalable. Low yields from cell disruption treatments can be explained by intrinsic resistance of Gram‐positive bacteria to cell disruption, enabled by their robust peptidoglycan cell wall. Past experience with Gram‐negative bacteria has shown genetic‐based manipulation of BEV biogenesis, via decoupling of outer and inner membrane integrity by genetic knockout of membrane integrity proteins (e.g., tolA), can generate orders of magnitude increased BEV yields.^[^ ^76^, ^77^, ^78^ ^]^ These genetic technologies have already been applied in industrial biomanufacturing of Gram‐negative BEVs for vaccine applications. However, homologous membrane integrity proteins do not exist in Gram‐positive bacteria. Thus, our approach was to apply genetic control over Gram‐positive BEV specific biogenesis mechanisms. Several, potentially overlapping, mechanisms of BEV biogenesis in Gram‐positive species have been proposed^[^ ^79^ ^]^: i) increased cytosolic turgor pressure,^[^ ^80^ ^]^ ii) outward membrane curvature, iii) decreased cross‐linking of the peptidoglycan cell wall,^[^ ^20^, ^81^ ^]^ and iv) membrane interactions with certain proteins, most notably the phenol soluble modulin peptides in *S. aureus*.^[^ ^80^ ^]^ We found engineering *L. plantarum* to express a recombinant enzyme known to remodel peptidoglycan cell wall cross‐linking resulted in 22‐fold increased BEV yields in 1/3 duration of time (66‐fold increased BEV production rate) compared to BEV yields from 24‐h culture of wild type *L. plantarum* (Figure 5). The hypervesiculating strain was cultured for only 8 h to cell densities around OD_600_ = ≈1.0, compared to 24 h conventional culture which produces cell densities of OD_600_ ≈4.0, and we did not normalize BEV yields by cell count. The system offers several advantages beyond yield and speed: i) inducible expression enables on‐demand BEV production at a defined bacterial growth stage, mitigating BEV heterogeneity from collection over wide spans of bacterial growth stages, ii) genetic modification avoids any additional processing steps, iii) standardized induction and timing may mitigate batch variability, and iv) the design is expected to be portable to other genetically tractable LAB chassis.

Finally, a key question asked in this study is whether hypervesiculating *L. plantarum* BEVs have therapeutic efficacy in murine colitis models. We found hypervesiculating BEVs reliably reduced several measures of disease severity in the acute DSS model, including key clinical trial endpoints of mucosal ulceration (Figure 6). Further investigation of therapeutic efficacy and mechanisms in other mouse models (e.g., chronic DSS, IL‐10 knockout, adoptive T cell transfer) will be useful in guiding clinical translation of hypervesiculating *L. plantarum* BEVs. Additionally, long‐term safety and biodistribution studies will be critical to determining the regulatory path and first in human dosing.

## 5 Conclusion

Overall, the data generated here support the conclusion that hypervesiculating *L. plantarum* BEVs are effective in the acute DSS murine model of IBD, potentially via mechanisms involving enhancement of mucosal tissue repair/protection and immunomodulation, especially with respect to oxidative tissue damage. As such, hypervesiculating *L. plantarum* BEVs are a promising potential novel IBD therapeutic that solves a key biomanufacturing limitation of EV therapeutics related to low production yields.

## Conflict of Interest

N.H.P., W.E.B., and S.M.J. have intellectual property interests (pending US patent application) related to extracellular vesicle technology.

## Supporting information



Supporting Information

Supporting Information

## Data Availability

The data that support the findings of this study are available from the corresponding author upon reasonable request.
