# Supplementary material for: Genetically‐Programmed Hypervesiculation of Lactiplantibacillus Plantarum Increases Production of Bacterial Extracellular Vesicles with Therapeutic Efficacy in a Preclinical Inflammatory Bowel Disease Model
Source: Adv Sci (Weinh). 2025 Nov 26;13(8):e12679. doi: 10.1002/advs.202512679 (PMC12884763; doi:10.1002/advs.202512679)
Supplement: Supplementary file 1 — Supporting Information [file ADVS-13-e12679-s002.docx]

**Genetically-programmed Hypervesiculation of *Lactiplantibacillus plantarum* Increases Production of Bacterial Extracellular Vesicles with Therapeutic Efficacy in a Preclinical Inflammatory Bowel Disease Model**

Nicholas H Pirolli, Daniel Levy, Alyssa Schledwitz, Natalia Sampaio Moura, Mitali Sarkar, Talia J. Solomon, Emily H. Powsner, Raith Nowak, Zuzanna Mamczarz, Christopher J. Bridgeman, Sulayman Khan, Nidhi Anne, Andrew Hui, Laura Reus, William E. Bentley, Jean-Pierre Raufman, Steven M. Jay

Supporting Information


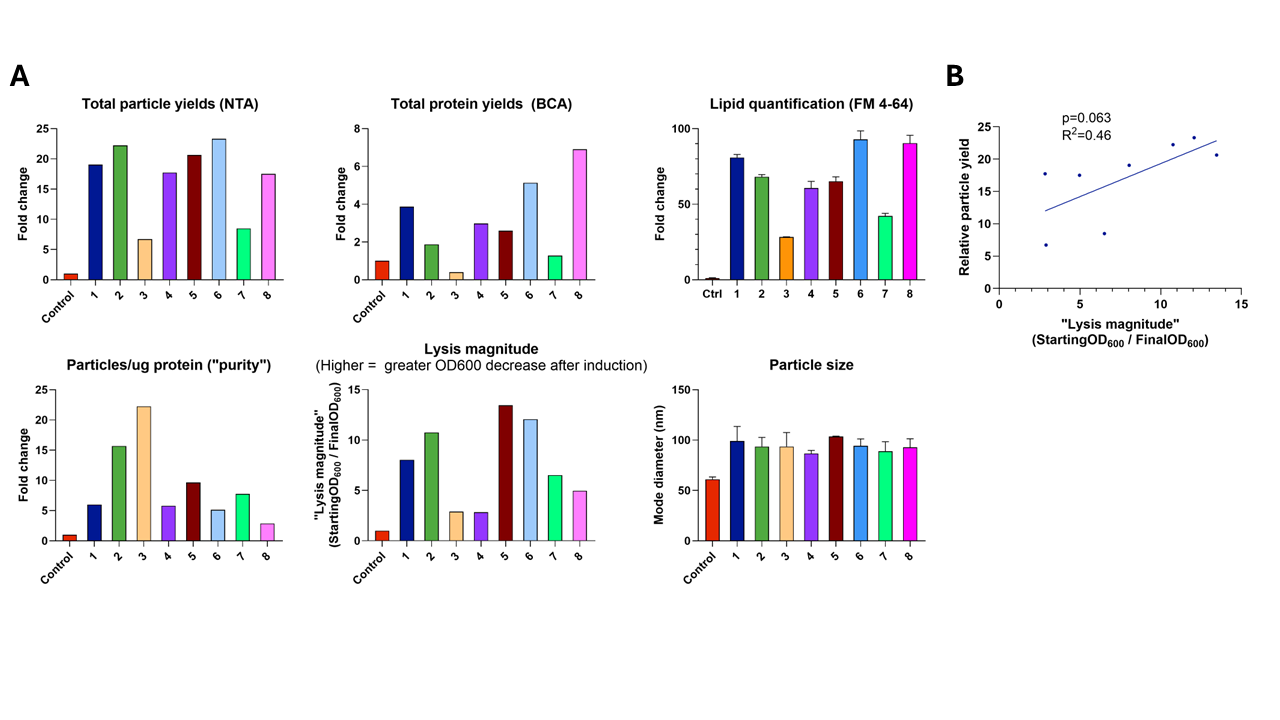
**Supplementary Figure 1. Hypervesiculating *L. plantarum* BEV yield weakly correlates with cell lysis. A)** Design of experiments (DOE) study evaluating yields, purity, particle size, and lysis magnitude (Starting OD_600_ / Final OD_600_ ) of hypervesiculating L plantarum tested under eight parameter combinations using 3-factor DOE assessing high/low values of inducer peptide, OD_600_ at induction, and duration after induction until BEV collection. **B)** Linear regression analysis of observed particle yield and ‘lysis magnitude’ in the eight different conditions. Lysis magnitude is calculated by the OD_600_ at time of induction (Starting) divided by the OD_600_ at time of BEV collection; higher values indicated greater reductions in cell density.


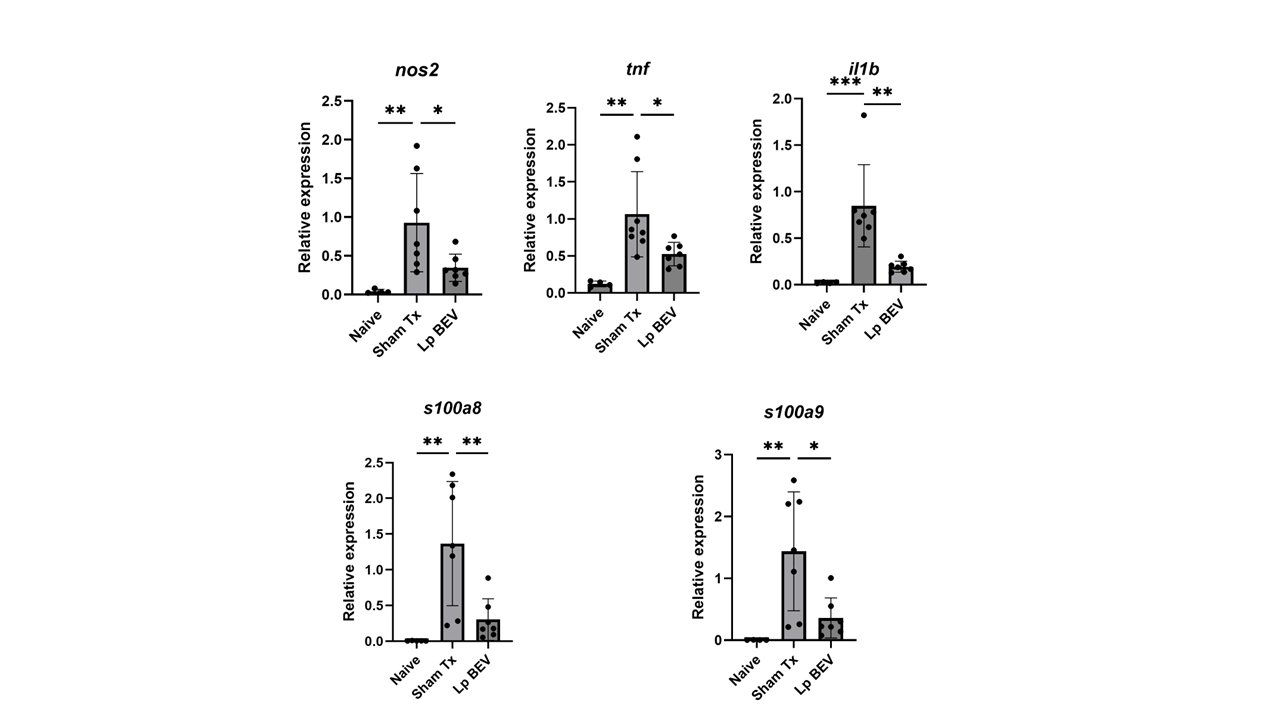


**Supplementary Figure 2. Gene expression in colon tissue from mice undergoing acute DSS-induced colitis.** cDNA from individual mice were analyzed by RT-qPCR to assess biologic variability in response to *L. plantarum* BEV (Lp BEV) or vehicle/Sham treatment. Statistical significance determined with one-way ANOVA and Tukey post hoc test * p<0.05, ** p<0.01, ***p<0.001, ****p<0.0001. Error bars ± SD.


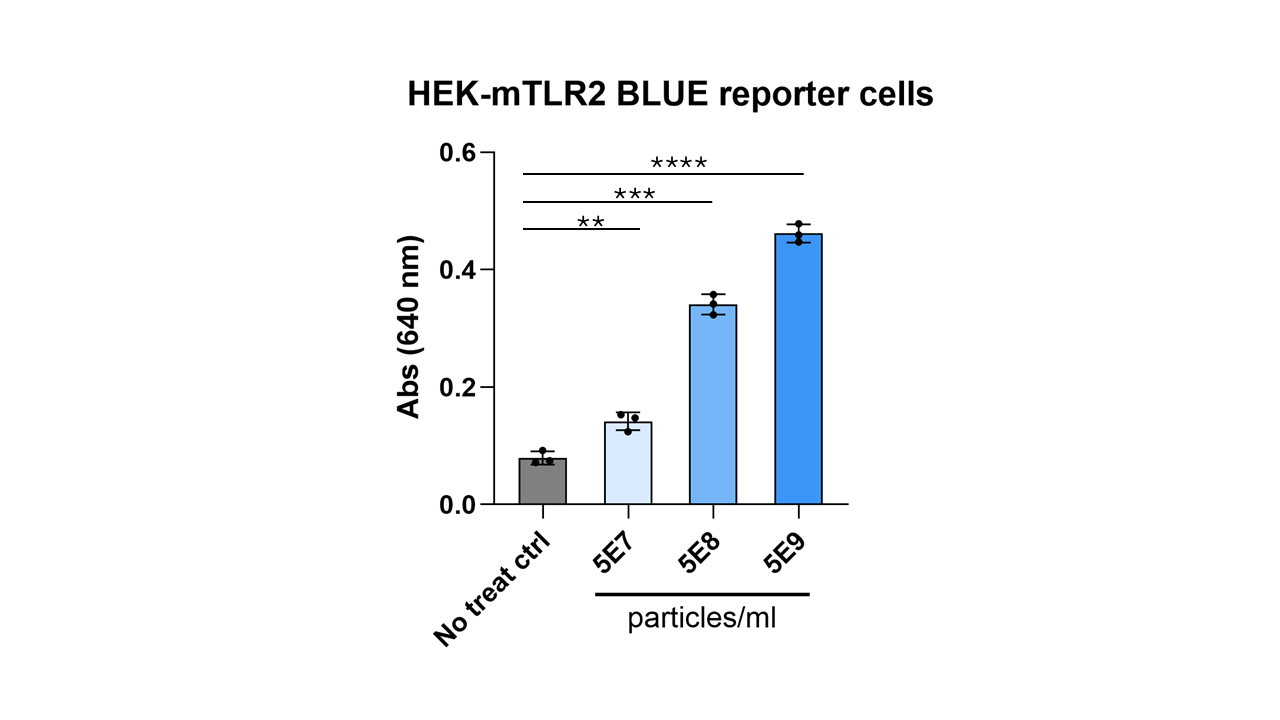


**Supplementary Figure 3. Hypervesiculating *L. plantarum* BEVs activate TLR2 signaling in HEK-Blue mTLR2 (HEK293T-derived) reporter cells.** Cells were treated with BEVs at the indicated concentrations; TLR2 activity was quantified 24 h later. Data are mean ± SD. One-way ANOVA with Dunnett’s test vs. vehicle; significance: * p < 0.05, ** p < 0.01, *** p < 0.001, **** p < 0.0001.
